# Influence of Drug Load on the Printability and Solid-State Properties of 3D-Printed Naproxen-Based Amorphous Solid Dispersion

**DOI:** 10.3390/molecules26154492

**Published:** 2021-07-26

**Authors:** Eric Ofosu Kissi, Robin Nilsson, Liebert Parreiras Nogueira, Anette Larsson, Ingunn Tho

**Affiliations:** 1 Department of Pharmacy, University of Oslo, P.O. Box, 1068 Blindern, 0316 Oslo, Norway; 2 Department of Chemistry and Chemical Engineering, Chalmers University of Technology, Kemivagen 10, 41296 Gothenburg, Sweden; robnils@chalmers.se (R.N.); anette.larsson@chalmers.se (A.L.); 3 Department of Biomaterials, Institute of Clinical Dentistry, University of Oslo, P.O. Box, 1109 Blindern, 0317 Oslo, Norway; l.p.nogueira@odont.uio.no

**Keywords:** 3D printing, additive manufacturing, fused deposition modelling, hot-melt extrusion, X-ray computed microtomography, glass solution

## Abstract

Fused deposition modelling-based 3D printing of pharmaceutical products is facing challenges like brittleness and printability of the drug-loaded hot-melt extruded filament feedstock and stabilization of the solid-state form of the drug in the final product. The aim of this study was to investigate the influence of the drug load on printability and physical stability. The poor glass former naproxen (NAP) was hot-melt extruded with Kollidon^®^ VA 64 at 10–30% *w*/*w* drug load. The extrudates (filaments) were characterised using differential scanning calorimetry (DSC), dynamic mechanical analysis (DMA), and thermogravimetric analysis (TGA). It was confirmed that an amorphous solid dispersion was formed. A temperature profile was developed based on the results from TGA, DSC, and DMA and temperatures used for 3D printing were selected from the profile. The 3D-printed tablets were characterised using DSC, X-ray computer microtomography (XµCT), and X-ray powder diffraction (XRPD). From the DSC and XRPD analysis, it was found that the drug in the 3D-printed tablets (20 and 30% NAP) was amorphous and remained amorphous after 23 weeks of storage (room temperature (RT), 37% relative humidity (RH)). This shows that adjusting the drug ratio can modulate the brittleness and improve printability without compromising the physical stability of the amorphous solid dispersion.

## 1. Introduction

It is becoming increasingly evident that more patient-specific medication is required owing to the uniqueness of patients. Patient-specific medication based on the patients’ genomics, biomarkers, clinical findings, and/or data from their lifestyle (e.g., information obtained using telemedicine technology) falls within the realm of personalised medicine [1,2,3]. For personalised medicine to work in practice, personalised pharmaceutical products need to be developed. One of the promising approaches to achieving this is the 3D printing of pharmaceutical products [4,5,6,7].

In pharmaceutical literature, fused deposition modelling (FDM) printing is combined with hot-melt extrusion (HME) to prepare the drug-loaded filament feedstock [8,9]. These manufacturing methods will often produce drugs in the amorphous form [10,11], which could offer benefits such as increased apparent solubility of poorly soluble drugs, and thereby increase their bioavailability [12]. For an amorphous drug, however, the drawback is their physical stability [13], and products from new manufacturing techniques such as 3D printing will not be immune to this. 3D printing based on FDM (combined with HME) involves heating and, therefore, mainly follows the thermodynamic pathway of producing amorphous drugs [14,15] and not the kinetic pathway involving mechanical disruption of crystals. Amorphous drug molecules lack a long-range molecular order and the molecules are arranged irregularly [16,17]. The molecules in an amorphous form, in addition to their short-range molecular order, are also very mobile at temperatures below their primary glass transition temperature (*T_g_*) [18]. This sort of mobility has been found to be the prime reason some amorphous drugs recrystallize when stored at room temperature [18]. Prevention of recrystallization of amorphous drugs (physical stabilization) can be achieved by forming a glass solution [17]. Glass solutions are single-phase amorphous systems and can be classified into non-polymeric glass solutions and polymeric glass solutions [19]. Non-polymeric glass solutions, such as co-amorphous systems, use low molecular weight compounds, e.g., amino acids, to stabilize the amorphous drug [20]. On the other hand, polymeric glass solutions stabilize amorphous systems via several factors including molecularly dispersing the drug in the molten polymeric matrix and thermodynamic solubilisation of the drug in the polymer [21,22]. This type of polymeric glass solution is termed amorphous solid dispersion (ASD) [20,21]. ASDs, aside from stabilizing an amorphous drug, can also improve apparent solubility as well as prevent recrystallization of the amorphous drugs during dissolution [23]. Improved drug dissolution rate, apparent solubility, and supersaturation are achieved firstly by solid-state conversion, improved wettability of the drug, and prevention of drug precipitation by the polymer used in the ASD [17].

In producing ASDs, HME is a widely used technique [24,25] and is a fusion-based technique involving melting, mixing, and extruding a crystalline drug and, usually, a thermoplastic polymer, between one or two co-rotating screws while heating the mixture above the *T_g_* of the polymer or above the melting point of the drug [8,26,27]. The molten polymer and/or drug then provides a medium to either solubilize or disperse the drug. The purpose of the screw(s) is to achieve mixing of the drug in the polymer and propel the mass towards the orifice where it is shaped during extrusion [8,26,27]. A successfully extruded product usually contains the drug in the amorphous form and exhibits a single *T_g_*. The advantages of using HME include solvent-free continuous processing and the ability to produce intermediate products, i.e., filaments for 3D printing.

3D printing of pharmaceuticals has gained substantial interest in academia and industry, and it is frequently suggested as a suitable platform for the production of personalised medicines [28,29]. 3D printing encompasses various printing techniques based on transforming a digital design (computer-aided design or scan) into a physical object and employs a layer-wise material deposition [5,30]. This enables new and customized product design, i.e., shape, size, geometry, internal channels, and so on; flexible dosing; and the possibility for drug combinations with varying complexity, which traditional pharmaceutical manufacturing does not easily allow [7,31]. This flexibility in design can be achieved by the material extrusion printing technique, which selectively deposits material from a nozzle [32]. Semi-solid extrusion and fused deposition modelling (FDM) are examples of printing platforms that use a nozzle to deposit material [8,10,33,34]. In pharmaceutical research, FDM is a preferred printing platform for developing and studying solid dosage forms [30,31,35,36]. FDM has several advantages and prime among them is the opportunity to couple FDM with the already frequently used HME technique to form a continuous manufacturing platform [8] and, the ability to produce an amorphous system for improving the apparent solubility of poorly soluble drugs.

HME and FDM-based 3D printing represent controlled means of producing amorphous drugs and ASDs. Once the ASD system has been formed, there is still the probability that the drug might recrystallize, hence the solid-state properties of the intermediate product (filament) and the final product (3D-printed tablets) need to be characterised and controlled.

A major issue in FDM-based 3D printing is the brittleness of the filaments; therefore, we show in this study the possibility of improving the printability of filaments simply by adjusting the drug load while maintaining the physical stability of the ASD. In this study, we have used HME to prepare filaments of Kollidon^®^ VA 64 (KVA64) and naproxen (poor glass former) at different concentrations. We performed solid-state characterisation on the resulting extrudates (i.e., the intermediate filaments) and printed tablets. We showed how various solid-state characterisation techniques can be combined to create a temperature profile supporting the selection of printing temperature and build plate temperature. The microstructure of the 3D-printed tablets correlating to successful printing was evaluated using X-ray computer microtomography (XµCT). The release of naproxen from the dosage form was assessed, and the storage stability with respect to the solid-state of the drug was followed for 23 weeks.

## 2. Results and Discussion

### 2.1. Development of Filaments by Hot-Melt Extrusion

An initial physical mixture containing 10–30% weight ratio (*w*/*w*) crystalline naproxen (NAP) and Kollidon^®^ VA 64 (KVA 64) was prepared and mixed at 160 °C using the HME and subsequently extruded into filaments at 140 °C. The selected temperatures were based on the *T_g_* (100 °C) and degradation temperature of KVA64 (230 °C) [37], and the melting point (155 °C) for naproxen. Filaments with a diameter of 2.75 ± 0.10 mm were produced. The filaments were of a glassy and transparent appearance, which may suggest that an amorphous system was achieved. A drug-free filament (0% NAP) was prepared as a reference.

Liu et al. have shown that a NAP-KVA64 system containing 48.5% *w*/*w* NAP will eventually crystallize [38]. A rapid screening test where 40% NAP was melted showed that the drug crystallized out upon cooling. Therefore, drug loads of 40% or higher were not studied.

### 2.2. Characterisation of Filaments

**Figure 1:**
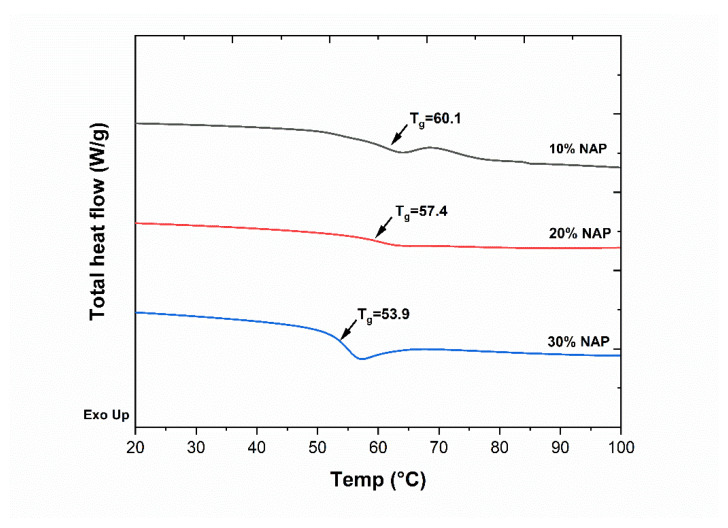
Differential scanning calorimetry (DSC) thermograms showing the decreasing glass transition temperatures (*T_g_*) of the filaments with increasing naproxen (NAP) content.

To check if an amorphous solid dispersion was formed, the *T_g_s* of the filaments were analysed using differential scanning calorimetry (DSC). The resulting DSC thermograms are shown in Figure 1, where a sigmoidal step change (endothermic event) can be seen for all the analysed filaments. In the figure, additional thermal events including enthalpy recovery and possibly evaporation of moisture from the 10% NAP can be observed. For 30% NAP, an enthalpy recovery is superimposed on the *T_g_* signal. The presence of a single *T_g_* for this binary mixture confirms that an amorphous solid dispersion was formed. It can also be observed that an increase in naproxen concentration results in a decrease in the *T_g_*. This indicates that naproxen plasticises KVA64. The conventional way to achieve plasticizing of the polymer would be to add low molecular substances, (e.g., PEG or triethyl citrate) or a low *T_g_* polymer (e.g., PEO), which act by lowering the *T_g_* of the mixture. Using the drug molecule to achieve plasticization rather than an additive will directly influence the drug loading. However, too high drug loading may lead to overplasticization and the drug forming a drug-rich phase, which may induce recrystallization of the amorphous drug upon storage, or precipitation and/or recrystallization of the amorphous drug during dissolution [39]. The addition of a plasticizer may also lead to the same stability issues. Adding up the plasticising effect of the drug itself with a conventional plasticizer may increase the risk of overplasticizing the polymer, thereby compromising the physical stability. As long as the required plasticization can be balanced with the physical stability and be provided by the drug molecule itself, this gives a simple formulation that is worth exploring.

### 2.3. Selection of Printing Parameters

**Figure 2:**
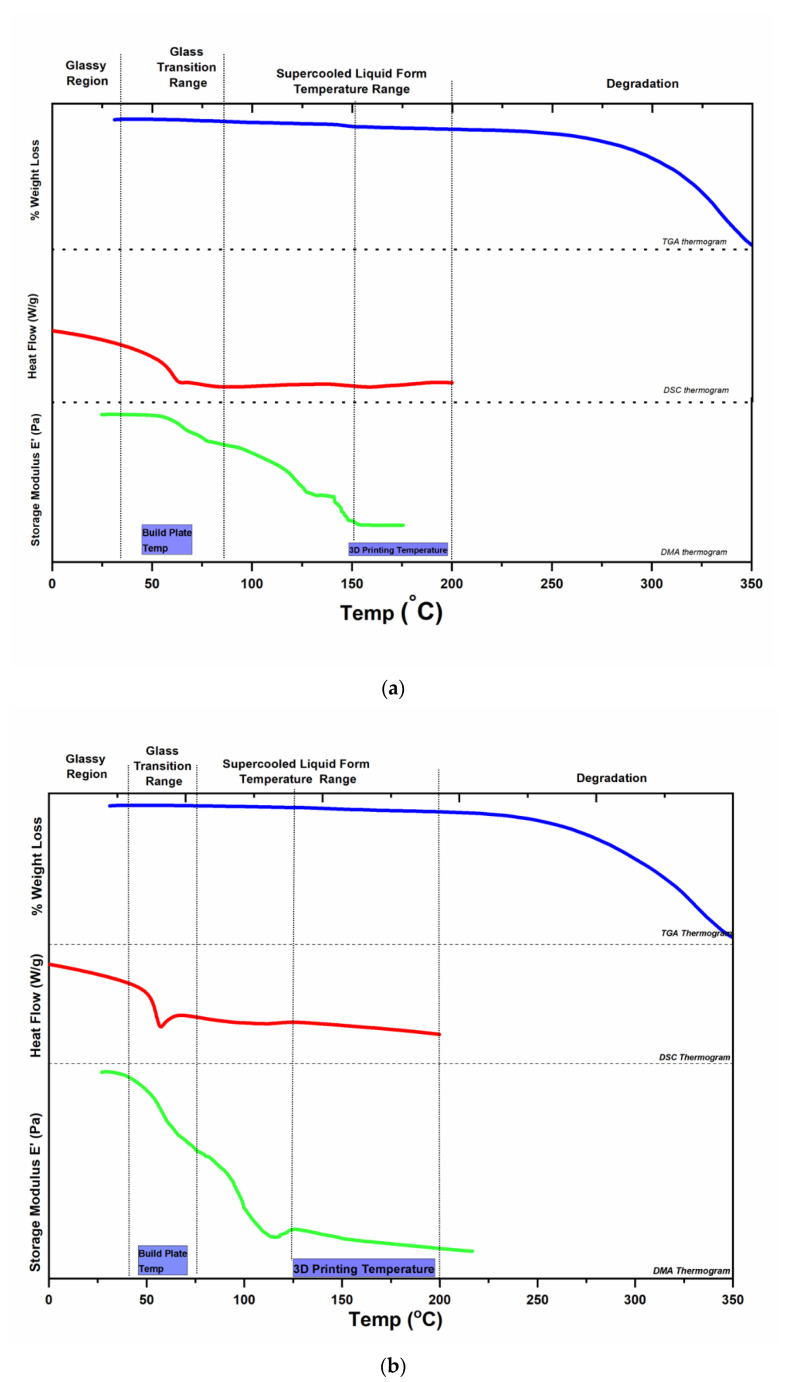
Temperature profile obtained by plotting TGA, DSC, and DMA thermograms of (**a**) 20% NAP and (**b**) 30% NAP filaments together for the selection of 3D printing temperature and build-plate temperature. Dash and dotted lines guide the eye to the various thermograms and temperature regions, respectively.

Before the filaments were printed into tablets, they were characterised for degradation with thermogravimetric analysis (TGA) and the transition temperatures were determined by dynamic mechanical analysis (DMA) and DSC. By plotting the TGA, DSC, and DMA thermograms together, a temperature profile of the filaments was obtained, see Figure 2. The temperature profile shows different regions; the temperature range where the filament is glassy, and the temperature range to expect solid-state transitions and the onset of degradation. From the glassy region, the beginning of cooperative molecular motions (*T_g_*) can be found. The *T_g_* signal is visible from 35 to 93 °C for 20% NAP and 32 to 81 °C for 30% NAP in the DSC and DMA thermograms (see Figure 2a,b). Just above the *T_g_*, the sample will assume a sticky rubbery state and the molecules can adhere to the build plate. As the cooperative molecular motions increase with increasing temperature, the viscosity will decrease, shown as a strong transition in the DMA thermograms between 95 and 135 °C and 81and 127 °C for 20% and 30% NAP, respectively. In the same temperature interval, a weak endothermic signal is seen in the DSC thermogram. This transition represents the flow of the material, and DMA is more sensitive to detect this and its corresponding transition compared with DSC. The second transition, which appears at a higher temperature, and a third transition, which appears between 135-155 °C for only 20% NAP, indicate that the 20% NAP filament does not have similar flow properties as that of 30% NAP filament. Using the DSC and DMA results, one can select temperatures from the midpoint of the signals, i.e., temperatures above 120 and 100 °C for 20% and 30% NAP, respectively, to determine printing temperatures. At the same time, the degradation temperature, determined by TGA, will determine the upper limit of the printing temperature. In this case, the upper limit was selected to be 200 °C, where the weight loss was around 2.9% and 2.3% for 20% and 30% NAP filaments, respectively.

Overall, the temperature range in which the ASD is not degraded and is in the supercooled liquid form will be suitable for selecting the temperature for 3D printing and build plate temperature. To further narrow down to a suitable printing temperature, it may be useful to consider the temperature range where there is no solid-state change, and in case the drug can crystallize, the melting point of the drug itself. This can be achieved by combining the thermal and the thermo-mechanical information as outlined. Such temperature profiles will also be relevant for selecting temperatures for further studying the rheological properties of filaments. In addition, this thermomechanical profile will reduce the assumptions made in selecting temperature ranges to ensure successful printing [40].

Based on the temperature profiles in Figure 2, a printing temperature of 160 °C and a build plate temperature of 60 °C were selected for 3D printing of the two types of filaments and the test for the differences in printing at different drug loadings.

### 2.4. 3D Printing

The printing of cylindrical tablets (diameter 8.0 mm, height 3.0 mm) was performed with a 100% infill density, no raft, and no blub. Filaments of approximately 5 cm were inserted into the print core of the Ultimaker 3 printer, and the tablet was 3D-printed individually one at a time. In this study, printability was defined as fabrication of the desired product without breakage of the filament, blockage of the nozzle, or any other printing hindrances [41]. Filaments containing 10% NAP were too brittle and could not be printed into tablets, whereas successful printing was achieved for filaments containing 20% and 30% NAP. The reference filament without drug was also too brittle to allow printing.

### 2.5. Characterisation of Printed Dosage Form with XµCT

XµCT analyses were performed on a representative selection of the 3D-printed tablets containing 20% and 30% NAP to determine the porosity of the 3D-printed tablets. This analytical tool is a non-invasive means to study the microstructure of the printed tablets, which might be linked to the placement of material by the nozzle [42,43].

**Figure 3:**
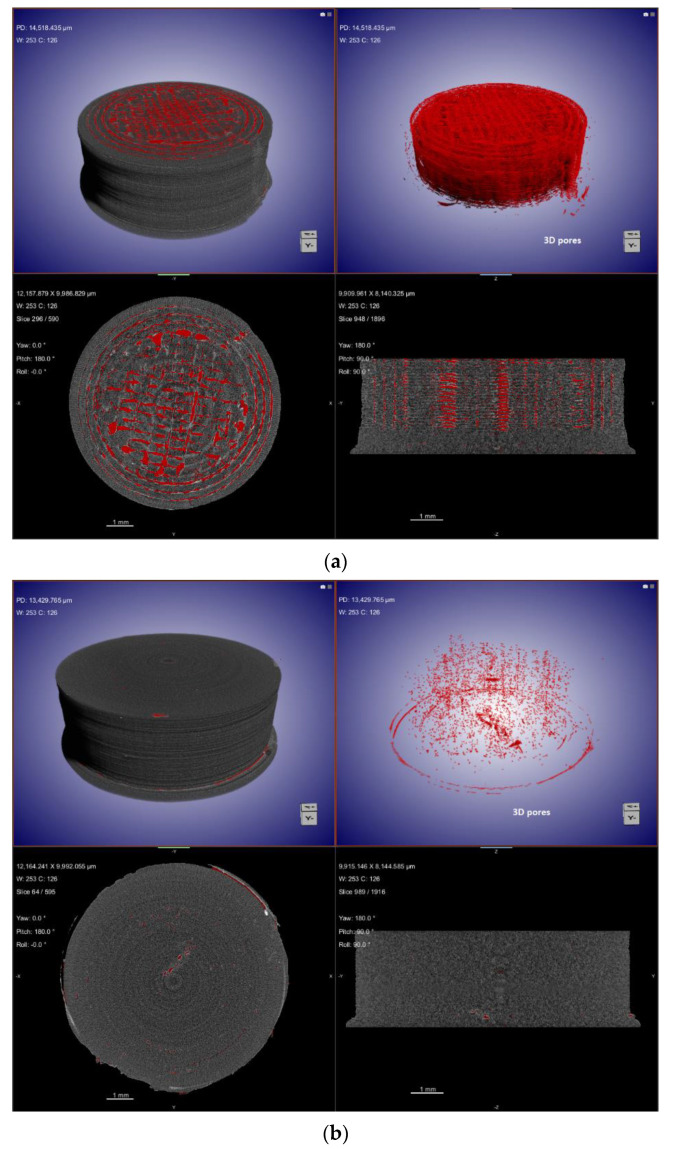
XµCT images showing material deposition and 3D pore distribution (red) in 3D-printed tablets containing (**a**) 20% NAP and (**b**) 30% NAP.

From the XµCT images in Figure 3, the 3D-printed tablet with 20% NAP had an average diameter of 8.06 mm and an average height of 3.11 mm. For tablet containing 30% NAP, an average height and diameter of 3.17 mm and 8.46 mm, respectively, were found (see Figure S1 and Table S1 in Supplementary Information). These values, however, did deviate from the CAD designed height of 3.0 mm and diameter of 8.0 mm. The deviation suggests that excess material was deposited [42]. The 3D porosity image of the tablet with 20% NAP, shown in Figure 3a, gives an indication of how the 3D printing was carried out; initially, a thick wall was created, followed by three inner walls and a grid infill. However, for the 30% NAP tablet, a denser structure was obtained, see Figure 3b. Both 20% and 30% NAP tablets were printed from the same CAD model and slicing file and with the same printer settings. The results from the XµCT analysis indicate that, during 3D printing of tablets containing 20% NAP, the nozzle may not place material in all areas as the design file states, whereas only a few air voids were created for the 30% NAP tablet, hence obtaining a denser structure. A dense structure without traces of printing wall and infill density pattern is an indication that material flowed well. These macroscopic differences in appearance were observed for all printed tablets from each of the two drug loadings; therefore, it was assumed to be explained by the different behaviour of the filaments under the selected conditions. Owing to the lower *Tg* determined for the filament with 30% NAP (Figure 1), the material will have lower viscosity over a longer time and can flow to fill the voids to a higher degree compared with the 20% NAP.

**Figure 4:**
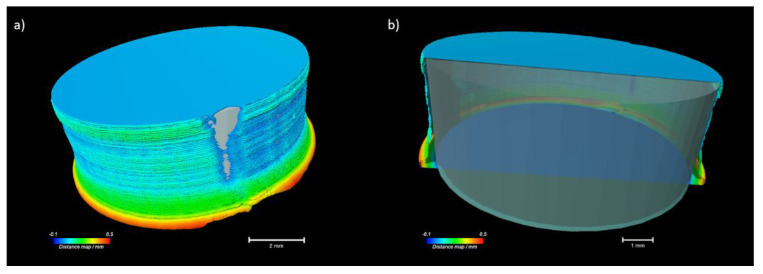
Comparison of the CAD model with the printed outer surface of the 3D-printed tablets containing 20% NAP. (**a**) Distance map comparing the surfaces of the CAD model and the 3D-printed tablet (colours show the displacement between the CAD model and the printed tablet; grey shows the CAD model). (**b**) Distance map (cropped halfway) and CAD model surface in lower opacity (grey).

The presence of air voids means the 3D-printed tablets are to some degree porous even though they were printed with 100% infill [44]. According to the CAD model, a dense structure should be expected. Figure 4 shows a comparison of the CAD model with the printed outer surface of the 3D-printed tablet containing 20% NAP. The distance map in Figure 4 confirms that the largest displacement between the CAD model and the printed tablet is in the bottom layer of the tablet sitting on the heated build plate.

**Figure 5:**
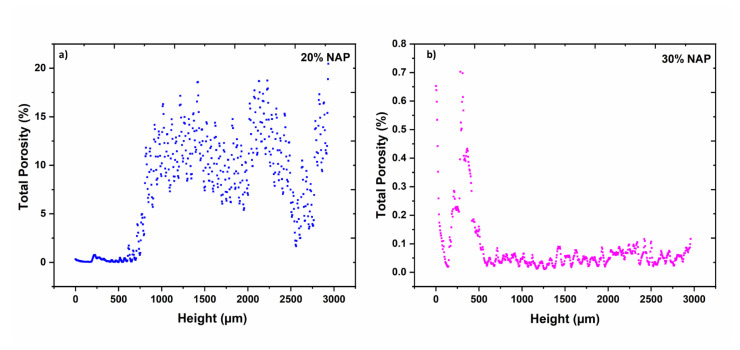
2D porosity of 3D-printed tablets containing (**a**) 20% NAP and (**b**) 30% NAP.

The porosity of the 3D-printed tablets was also characterised as a function of the height (2D porosity) and is presented in Figure 5. For the 20% NAP tablet, a dense structure was observed up to approximately 0.5 mm (see Figure 5a), and from then, air voids or pores can be noticed. For the 30% NAP tablet, it seems that the air void begins to form from the first layer to around 0.5 mm, see Figure 5b. However, it should be noted that the total porosity in this region was below 0.8%, which is in the same order of magnitude as the porosity in the 20% NAP in the bottom part of the printed tablet. The porosity decreased further in the 30% NAP tablet.

The total porosity (see Table 1) of 3D-printed tablets was found to be 7.79% and 0.09% for 3D-printed tablets with 20% and 30% NAP, respectively. The similarity of the pore profiles on the base of the printed tablets, i.e., up to 0.5 mm, lies in the fact that the tablets are dense at this region of the tablet, which is the part of the tablet sitting at the heated build-plate. This indicates that the material flows more and closes potential pores during printing, and it is a typical finding for FDM-printed items. The higher porosity observed for 20% NAP might be related to the suboptimal printing temperature for this combination, with slightly too high viscosity of the molten material. This impacts the flow of the material from the nozzle and results in a more uneven deposition.

### 2.6. Solid-State Properties of the Printed Dosage Form

**Figure 6:**
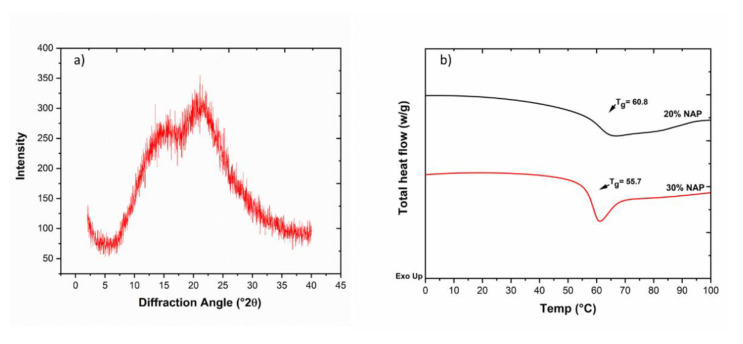
(**a**) Diffractogram from the 3D-printed tablets containing 30% NAP; (**b**) DSC thermogram of tablets containing 20% and 30% NAP.

The solid-state form of the printed tablets was studied with X-ray powder diffraction (XRPD) and DSC, and the results are shown in Figure 6. It can be seen that the diffractogram from the XRPD shows a halo, which is an indication of amorphousness. Tablets from both filament types showed the same diffractogram. The thermograms in Figure 6b show that, after 3D printing, the dosage forms have *T_g_*s, which are similar to that of their filaments and might be an initial confirmation that no degradation occurred during the 3D printing. In addition, single *T_g_*s observed is an indication of a single-phase amorphous system i.e., ASD [17].

### 2.7. Drug Release

**Figure 7:**
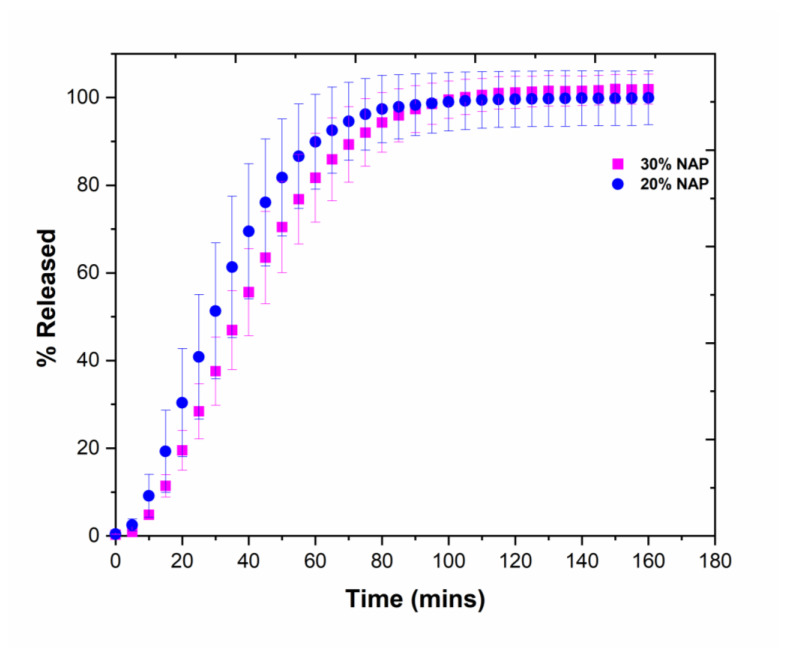
Release profile of NAP from 3D-printed tablets containing the 20% and 30% NAP determined in phosphate buffer pH 7.4 (*n* = 3).

The release profile of naproxen was studied in a standard dissolution test using phosphate buffer pH 7.4 as a dissolution medium. The 3D-printed tablets containing 20% NAP weighted 173.4 ± 3.4 mg, equivalent to approximately 35 mg NAP, while 30% NAP tablets weighted 196.5 ± 5.3 mg, which has a dose equivalence of approximately 60 mg NAP. The percentage drug released as a function of time is shown in Figure 7. The variation between the tested tablets reflects the variation in mass between the tablets and thus also the drug content. With approximately 80% released after 50 min for 20% NAP and 60 min for 30% NAP, the percentage drug release was slightly faster for the 20% NAP tablets even though the difference was not statistically significant (*p* > 0.05) owing to variation between the individual tablets. The release rates correlate with XuCT analysis, which showed that 20% NAP has a higher total porosity as compared with 30% NAP (i.e., 7.79% versus 0.09%). The higher the porosity, the faster the water ingress into the water-soluble matrix of the tablets, leading to faster drug release. The release from both types of 3DP tablet formulations was slightly slower than “conventional-release tablets” according to the Ph.Eur., which states that 80% should be released within 45 min (Ph. Eur. 5.17.1). Even though the polymer matrix is hydrophilic and water-soluble, the slower release rate is probably because of the fact that the water ingress was impeded owing to very dense tablets with low porosity, as discussed above, combined with the low aqueous solubility of the drug (15.8 mg/L).

Scrutinizing the absolute amount of drug released per time unit, the formulation with the highest drug load showed faster release, which can be explained by the higher concentration gradient driving the diffusion of the drug from the matrix (e.g., 30 mg NAP released in approximately 55 min from 20% NAP and approximately 35 min from 30% NAP 3DP tablets). Therapeutically, NAP is interesting both as conventional or immediate release tablets and as sustained-release tablets. Selecting printing parameters that would result in a less dense tablet could be a means to increase the release rate [45], whereas to obtain slower and more sustained release, it might be necessary to combine the polymer with a less soluble polymer or change to a different polymer [12]. In addition, gastro-resistant tablets may be achieved by selecting pH-responsive polymers, such as hydroxypropyl methylcellulose acetate succinate and methacrylic acid-ethyl acrylate copolymer [46].

### 2.8. Physical Stability of Printed Tablets

**Figure 8:**
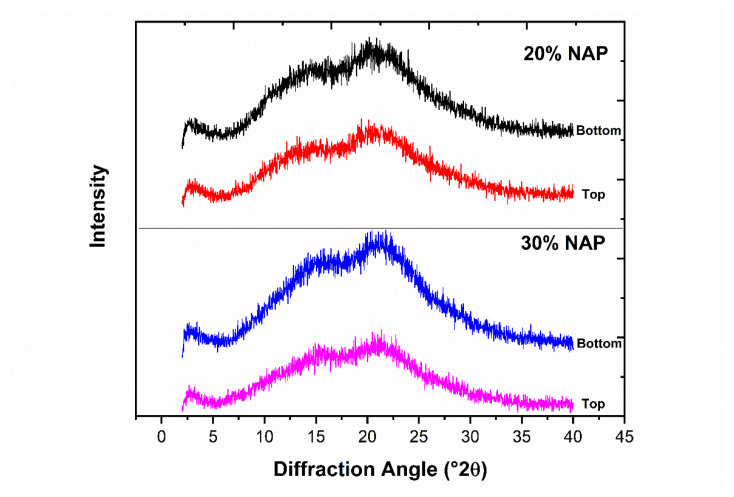
Diffractograms from the top and bottom sides for the 3D-printed tablets after 23 weeks of storage at 37% relative humidity (RH) and 298 K.

NAP cannot be made amorphous alone via the thermodynamic or mechanical pathway [47,48]; however, amorphization can be achieved by formulating it as an ASD. The physical instability of amorphous NAP means it can revert to its crystalline form even when formulated as an ASD. We, therefore, stored the 3D-printed tablets containing 20% and 30% NAP at ambient conditions in a desiccator for 23 weeks and characterised their solid-state thereafter. The relative humidity in the desiccator during the storage under ambient conditions was measured at 37% RH. The diffractograms (Figure 8) from the top and bottom sides of the 3D-printed tablets for the two different drug loadings show for all four different cases a halo diffractogram after 23 weeks. The obtained results are similar to the diffractogram of the freshly 3D-printed tablets (Figure 6). X-ray is known to be a powerful method for detecting low crystalline content in amorphous materials [49], e.g., in an amorphous/crystalline sucrose mixture, the limit of detection was found to be 0.9% *w*/*w* and the limit of quantification was 1.8% *w*/*w* [50]. This indicates that there was no or very low, below the detection limit, solid state change and that the dosage forms retained their amorphous forms even after exposure to ambient temperature and moisture. The absence of diffraction peaks also indicates that naproxen at a concentration of 20% and 30% is thermodynamically soluble in KVA 64 and the drug will not recrystallize during these storage conditions [51].

## 3. Materials and Methods

### 3.1. Materials

Naproxen (Mw 230.25 g/mol, *T_m_* 155 °C) was purchased from MP Biomedicals, Fisher Scientific, Sweden and Kollidon^®^ VA 64 (KVA64) was generously donated by BASF, Ludwigshafen, Germany.

### 3.2. Hot-Melt Extrusion

NAP and KVA64 were weighed and thoroughly mixed to form a physical mixture containing 10, 20, and 30% NAP. Then, 5 g batches of each of the drug-polymers blends were extruded using a lab-scale twin-screw compounder (XPLORE, DSM, Urmonderbaan, The Netherlands). All six heating zones were preheated to 160 °C and the batch of 5 g was molten, mixed, transported, and subsequently extruded at 140 °C through the die (diameter 2.5 mm) into filaments.

### 3.3. 3D Printing

Autodesk^®^ Fusion 360^TM^ v.2.0.5688 software was used to design a cylindrical tablet structure with a height of 3.0 mm and a diameter of 8.0 mm. The generated computer-aided design files were converted into stereolithographic (STL) files for further slicing in Ultimaker Cura software. 3D-printed tablets were printed on an FDM printer (Ultimaker 3 printer Zaltbommel, The Netherlands). The selected printing parameters were 100% infill density, 0.05 mm layer height, printing temperature of 160 °C, and a build plate temperature of 60 °C.

The resulting printed cylindrical tablets containing either 20% or 30% NAP were stored in a desiccator at 37% RH at 25 °C for further analysis.

### 3.4. Thermogravimetric Analysis

Thermal degradation of the filaments was performed on a TGA/DSC 3 + STARe System (Mettler-Toledo, Greifensee, Switzerland). Approximately 10 mg of filament containing 20% and 30% NAP was placed in Al crucibles. The change in weight was analysed at a heating rate of 10 °C/min from 25 to 350 °C. Data analyses were performed with STARe Evaluation v.16.10 software.

### 3.5. Differential Scanning Calorimetry

The thermal behaviour of the filaments and the 3D-printed tablets was analysed with DSC822 (Mettler-Toledo, Greifensee, Switzerland). Approximately 4 mg of sample was placed in a 4 µL Al crucible and sealed with a perforated lid. The thermal behaviour of the samples was measured at a heating rate of 10 °C/min from 0 to 200 °C. The *T_g_* was analysed from the midpoint of the sigmodal change in the heat capacity. Three replicates were performed and data analyses were performed with STARe Evaluation v.16.10 Software.

### 3.6. Dynamic Mechanical Analysis

Filaments containing 20 and 30% NAP were milled using a mortar and pestle. The resulting powder sample was loaded into a stainless steel powder sample holder and clamped into a 35 mm dual cantilever. Duplicate DMA scans were measured on a DMA Q 800 (TA Instruments−Waters LLC, New Castle, DE, USA) using an amplitude of 20 µm, a frequency of 1 Hz, and a heating rate of 3 °C/min from 25 to 220 °C. Data analysis was performed using TA Universal analysis software.

### 3.7. X-ray Powder Diffraction

The solid-state analysis was performed with a Bruker D8 Discovery X-ray powder diffractometer (Kontich, Belgium). Cu K radiation (λ = 1.5406 Å) was generated using an acceleration voltage of 40 kV and a current of 50 mA. The 3DP tablet was mounted on adhesive material and the diffraction pattern was recorded from 2 to 40° 2theta.

### 3.8. X-ray Computer Tomography

The samples were scanned using the Skyscan 1172 microCT system from Bruker (Kontich, Belgium). The projections were acquired with a final isotropic voxel size of 5.0 µm, at camera binning 2 × 2 of a CCD camera (11 Mpixel CCD detector), 44 kV accelerating voltage, and 200 μA current, with no physical filter placed in front of the beam. During the acquisition, the sample was rotated by 360° about its vertical axis at a step size of 0.79°, with an exposure time of 240 ms per projection, taking an average of three frames for reducing the noise. Cross-sectional images were reconstructed with NRecon v.1.7.1.0 software (Bruker) with a filtered back-projection algorithm and the following parameters: ring artefact correction of 9, beam hardening correction of 40%, and no smoothing. The porosity analysis was performed using CT Analyser (CTAn 1.18.1.0+, Bruker). Pores with dimensions smaller than a threshold of 4 × 4 × 4 pixels were excluded from the analysis. The 3D images were rendered using Dragonfly software v. 2021.1 (Object Research Systems (ORS) Inc, Montreal, QC, Canada).

### 3.9. Physical Stability

The 3D-printed tablets were kept at ambient conditions in a desiccator. The humidity during storage was at 37% RH at room temperature (25 °C). XRPD analyses were performed from both sides of the 3DP tablets after 23 weeks.

### 3.10. Drug Release

The drug release was determined in vitro for the tablets using USP apparatus II from Varian VK 7025 coupled with Varian Cary UV–visible spectrophotometer. The tablets containing 30% NAP were placed in 1000 mL phosphate buffer (pH 7.4, 37 °C, 50 rpm). Aliquots were pumped by peristaltic pumps into cuvettes after every five minutes and analysed at 332 nm. Three independent tablets were analysed. The mass of the individual tablets was determined prior to testing.

## 4. Conclusions

By formulating NAP with KVA 64, we have been able to hot-melt extrude this formulation into filaments suitable for FDM printing of tablets. The extrudates were found to be an amorphous solid dispersion. Thermomechanical analysis, i.e., a combination of TGA, DSC, and DMA, was used to develop a temperature profile from which a suiTable 3D printing temperature was selected. Filaments containing 20% and 30% NAP were suitable for 3D printing, whereas 0% NAP and 10% NAP resulted in filaments that were too brittle. Microstructure analysis of tablets containing 20% NAP showed more pores or voids, which could be an indication that the 3D printer did not deposit material in all required space. Overall, the 3D-printed tablets were found to be physically stable and to retain their amorphous form for 23 weeks. The dissolution profile of the 3D-printed tablets showed a relatively fast release with 80% released within approximately 50 min for 20% NAP and approximately 60 min for 30% NAP. This work shows how thermal and thermomechanical analysis can be used to build temperature profiles for selecting temperature-related parameters for FDM-based 3D printing. In addition, by increasing the drug load, it is possible to 3D print pharmaceuticals without compromising the physical stability of the amorphous solid dispersion.

## Figures and Tables

**Table 1:** Physical average dimensions and porosities determined by XµCT of representative 3D-printed tablets containing 20% and 30% NAP, respectively.

Parameter	Unit	20% NAP	30% NAP
Height of tablet ^1^	mm	3.11	3.17
Mean diameter ^1^	mm	8.06	8.46
Open porosity ^1^	%	6.67	0.02
Closed porosity ^1^	%	1.12	0.07
Total porosity	%	7.79	0.09

^1^ See Supplementary Materials (Table S1 and Figure S1) for information and images defining the respective measurements.

## Data Availability

Not applicable.

## Supplementary Materials

The following are available online. Figure S1: Regions for measuring diameter and height measurement from XµCT images of the 3D-printed tablets; Table S1: Definitions of porosity terms used in this study. Refer also to Figure S1.

## Sample Availability

Samples of the compounds are not available from the authors.
