# Supplementary material for: Influence of Drug Load on the Printability and Solid-State Properties of 3D-Printed Naproxen-Based Amorphous Solid Dispersion
Source: Molecules. 2021 Jul 26;26(15):4492. doi: 10.3390/molecules26154492 (PMC8347219; doi:10.3390/molecules26154492)
Supplement: Supplementary file 1 [file molecules-26-04492-s001.zip › molecules-1280937-supplementary.pdf]

molecules

MDPI

Supplementary Materials

Influence of Drug Load on the Printability and Solid-State Properties of 3D-Printed Naproxen-Based Amorphous Solid Dispersion

Eric Ofosu Kissi [1,*], Robin Nilsson [2], Liebert Parreiras Nogueira [3], Anette Larsson [2], and Ingunn Tho [1,*]

[1] Department of Pharmacy, University of Oslo, P.O. Box, 1068 Blindern, 0316 Oslo, Norway
[2] Department of Chemistry and Chemical Engineering, Chalmers University of Technology, Kemivagen 10, 41296 Gothenburg, Sweden; robnils@chalmers.se (R.N.); anette.larsson@chalmers.se (A.L.)
[3] Department of Biomaterials, Institute of Clinical Dentistry, University of Oslo, P.O. Box, 1109 Blindern, 0317 Oslo, Norway; l.p.nogueira@odont.uio.no
* Correspondence: eric.of.kissi@gmail.com (E.O.K.); ingunn.tho@farmasi.uio.no (I.T.); Tel.: +47-2284-4455

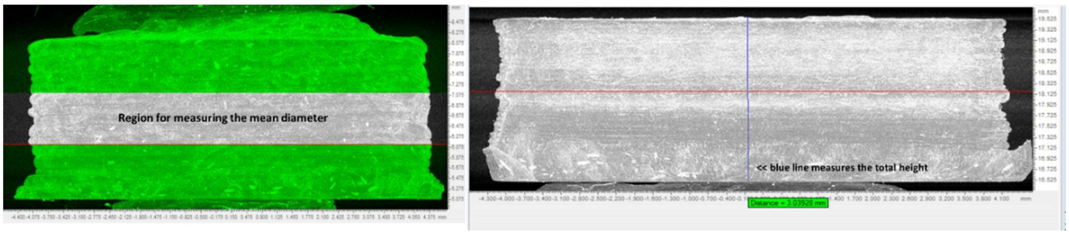

Figure S1: Regions for measuring diameter, and height measurement from XμCT images of the 3D printed tablets.

Table S1. Definitions of porosity terms used in this study. Refer also to Figure S1.

Term	Definition
Mean diameter	Mean diameter was calculated from the measurement of the cross-sectional area, taking the average of 1.0 mm of material in the middle of the tablet, having the tablet cross-section considered to be a circle
Open porosity	Pores connected to the outside of the tablet, either on the sides or on the top and bottom
Closed porosity	Voids inside the tablet, which do not have access to the surface of the tablet
2D porosity	This is a slice-based measurement of the voids in the material and is based on the count of pixels pertaining to voids. The measurement is performed in each single slice of 5.0 μm thickness. It must not be confused with 3D porosity, which takes into account the connectivity of the voids throughout the space.
